# Preparation, Thermal Regulation, and Energy Storage Properties of n-hexadecane@polymethyl Methacrylate Microcapsule–Cement Composite Phase Change Materials

**DOI:** 10.3390/polym18131609

**Published:** 2026-06-28

**Authors:** Houqi Zhu, Jianmin Ma, Xiaoxiao Xing, Heng Wang, Lixian Sun, Cuili Xiang, Yongjin Zou

**Affiliations:** 1 College of Materials Science and Engineering, Guilin University of Electronic Technology, Guilin 541004, China; 2 Guilin Huayue Environmental Protection Technology Co., Ltd., Guilin 541805, China

**Keywords:** building materials, phase change materials, microcapsules, energy storage

## Abstract

With the continuous growth in global energy consumption and the increase in the proportion of energy use attributed to buildings, the development of highly efficient and energy-saving building materials has become necessary for reducing energy demands and greenhouse gas emissions. Phase change materials (PCMs) exhibit great potential for enhancing the thermal inertia of buildings owing to their ability to efficiently absorb and release latent heat during phase transitions. In this study n-hexadecane@ polymethyl methacrylate (16-MMWS-K) microcapsules (where “@” denotes the core-shell encapsulation structure) with a crosslinked structure were successfully prepared via emulsion polymerization, using n-hexadecane as the core material and polymethyl methacrylate as the shell. The prepared microcapsules were incorporated into a cement matrix to fabricate a phase-change energy-storage composite material. The morphology, structure, and thermal properties of the microcapsules, as well as their effects on the thermal and mechanical performance of the cement composites, were systematically investigated. The prepared 16-MMWS-K microcapsules exhibited a well-defined core–shell structure, excellent thermal stability, and a suitable phase-change temperature. Increasing the microcapsule content significantly enhanced the thermal energy storage capacity of the cement composites, reduced thermal conductivity, improved hydrophobicity, and demonstrated effective temperature regulation in building simulation experiments. This study provides both theoretical insight and experimental evidence supporting the practical application of 16-MMWS-K microcapsules in cement composites. The 28-day compressive strength (51.7 MPa) remains acceptable despite higher porosity and slight strength reduction.

## 1. Introduction

The continuous growth in global energy consumption and the urgent need to reduce greenhouse gas emissions have positioned the building sector as a critical area for improvement, as it accounts for a significant portion of total energy use [1,2,3]. Latent heat storage technology, which leverages the efficient absorption and release of heat during the phase transitions of phase change materials (PCMs), offers an innovative solution for enhancing building thermal inertia and mitigating indoor temperature fluctuations [4,5,6]. Microencapsulated phase change materials (MEPCMs) encapsulate PCMs within polymer shells. This approach effectively addresses key limitations of traditional PCMs, such as leakage, high corrosivity, and poor compatibility with host materials, while optimizing thermal stability and mechanical adaptability by regulating the shell composition and structure. Consequently, MEPCMs are particularly suitable for integration into building material systems, such as cement-based matrices [7,8,9,10,11].

PCMs are mainly of the following types: organic, inorganic, and eutectic [12]. Among organic PCMs, paraffin waxes and n-alkanes offer significant advantages, including high latent heat of phase change, excellent chemical and thermal stability, low saturated vapor pressure, and low cost, making them a primary focus of research and development [13]. Microencapsulation technology has been widely adopted to overcome leakage of PCMs in the liquid phase [14], mitigate their corrosiveness, and enhance their safety and convenience [15]. This technique produces a core–shell structure in which the PCM is encapsulated within a polymer shell, forming a MEPCM. Preparation methods for MEPCMs can be broadly categorized into physical and chemical methods [15]. Physical methods include spray drying [16], fluidized bed processes [1], and solvent evaporation [16], whereas chemical methods include in situ polymerization [17,18,19,20], such as interfacial polycondensation, suspension polymerization, and emulsion polymerization, as well as simple or composite condensation processes [21].

The practical performance of MEPCMs is jointly determined by the properties of the PCM core and polymer shell [22,23,24]. To optimize performance, various encapsulation techniques using different polymeric shell materials have been explored. Wang et al. prepared stearic acid/polymethyl methacrylate nanocapsules, with excellent thermal properties, using sonochemical methods [25]. Önder et al. utilized an ecofriendly approach for shell material and successfully encapsulated three PCMs—n-n-hexadecane, n-octadecane, and n-nonadecane—using a composite coagulation method using a natural biodegradable polymer (gum Arabic-gelatin) shell [26]. Platte et al. synthesized microcapsules with alkaline hydrate salts as cores via surface Michael addition polymerization between acrylates and thiols [27]. Polyurea microcapsules encapsulating n-octadecane with high monodispersity were fabricated using tubular microfluidic technology [27]. Regarding the size effects of microcapsules, Paula et al. demonstrated through studies on n-docosane/polyurethane capsules that reducing capsule size and increasing the specific surface area effectively enhanced the melting and crystallization enthalpies [28]. Additionally, Fortuniak et al. employed co-emulsification technology to encapsulate n-eicosane within a siloxane polymer shell, achieving improved thermal performance and structural stability [29].

Among the numerous PCMs, n-hexadecane is considered an ideal core material for latent heat storage applications in buildings because of its suitable phase transition temperature that closely aligns with indoor comfort ranges, as well as its excellent chemical stability and low cost [30,31]. PMMA is a commonly used microcapsule shell material owing to its good film-forming ability, thermal stability, and chemical inertness, providing a reliable physical barrier for the n-hexadecane core and effectively reducing leakage risks during preparation and service in cement-based systems [32,33]. However, two major challenges remain regarding the application of 16-MMWS-K microcapsules in cement matrices. The highly alkaline environment and exothermic hydration process of cement may disrupt the microcapsule shell structure, leading to premature leakage of the core material and compromised energy storage performance [34,35,36]. However, the interfacial bonding state between the microcapsules and cement matrix, along with the influence of microcapsule dosage on the mechanical properties of cement composites, directly determine their feasibility in practical construction applications [37,38,39]. Currently, research on the synergistic mechanism between n-hexadecane-PMMA microcapsules and cement matrices remains unsystematic. In particular, the effects of shell modification on thermal stability of microcapsules in cement matrices [40,41,42], as well as the balanced relationship between microcapsule loading and the thermomechanical properties of cement composites, have not yet been clearly resolved.

To address these issues, an emulsion polymerization method was employed in this study. n-hexadecane was used as the PCM for the core, while methyl methacrylate monomer, together with the crosslinking agent N,N-methylenebisacrylamide, served as the shell matrix. 16-MMWS-K microcapsules with a crosslinked structure (designated as 16-MMWS-K) were successfully synthesized via potassium-persulfate-initiated in situ radical polymerization, forming a stable PMMA-based copolymer shell on the surface of n-hexadecane droplets. The prepared microcapsules were incorporated into cement matrices at various concentrations to fabricate phase-change energy-storage cement composites. The findings indicate that the developed 16-MMWS-K microcapsules exhibit promising potential for building energy conservation by enhancing the thermal inertia of building envelopes and reducing heating and cooling energy consumption.To address these issues, this study aims to synthesize crosslinked PMMA-based microcapsules with enhanced shell robustness against alkaline cement environments, systematically evaluate the effect of microcapsule loading (0–30 wt%) on the thermal storage, thermal insulation, hydrophobicity, and mechanical properties of cement composites, and clarify the trade-off between thermal enhancement and mechanical degradation. The originality lies in the use of N,N-methylenebisacrylamide as a crosslinker to improve shell integrity, and in providing a comprehensive structure-property relationship for such composites, which has not been previously reported in a single study.

## 2. Experimental Section

### 2.1. Materials

n-hexadecane (CAS: 544-76-3), used as the core material for microcapsule phase change materials; methyl methacrylate (CAS: 80-62-6), N, N,N-Methylenebisacrylamide (CAS: 110-26-9), used as a copolymer monomer for microcapsule wall material; sodium dodecyl sulfate (CAS: 151-21-3), as an emulsifier; potassium persulfate (CAS: 7727-21-1), as a polymerization initiator; glacial acetic acid (CAS: 64-19-7), for adjusting the pH of the aqueous phase; cement sourced from Guilin Lushan New Building Materials Co., Ltd. (industrial grade, Guilin, China), used as the matrix material for cement composites; all aqueous phases used during experiments were deionized water (18.2 MΩ·cm).

### 2.2. Preparation

#### 2.2.1. Preparation of 16-MMWS-K Microcapsules

**Figure 1:**
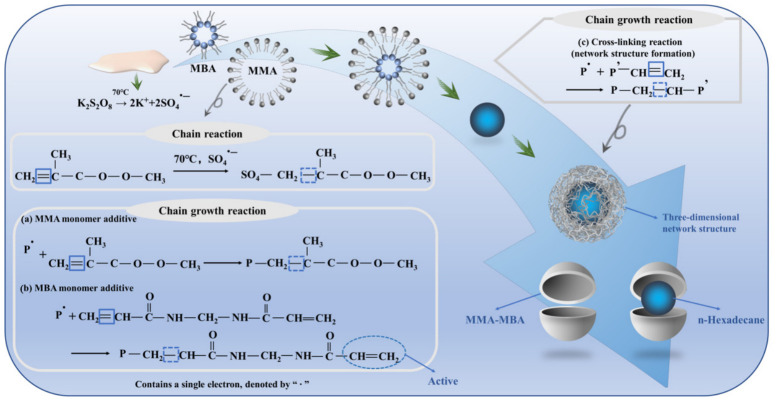
Flowchart showing the preparation process of 16-MMWS-K microcapsules, (**a**) MMA monomer additive, (**b**) MBA monomer additive, (**c**) cross-linking reaction.

Microcapsule PCMs with n-hexadecane as the core material and poly(methyl methacrylate-N,N-methylenebisacrylamide) as the shell layer were prepared via emulsion polymerization. The preparation process includes oil- and water-phase preparation, emulsification, polymerization, and post-treatment steps, as illustrated in Figure 1.

Oil-phase preparation: n-hexadecane, methyl methacrylate (MMA), and N,N-methylenebisacrylamide (MBA) were mixed in a mass ratio of 20:40:1 and stirred at 40 °C and 500 rpm for 30 min to obtain a homogeneous oil phase solution.

Aqueous-phase preparation: sodium dodecyl sulfate (SDS) was added to deionized water at a mass ratio of 1:200 and the solution was stirred at 300 rpm for 25–35 min. Glacial acetic acid was then added dropwise to adjust the pH to 3, forming the aqueous phase solution.

Emulsification: The oil phase was slowly added to the aqueous phase, followed by high-shear emulsification at 40 °C and 800 rpm for 20 min to obtain a stable oil-in-water emulsion.

Polymerization and post-treatment: Potassium persulfate, used as a water-soluble initiator at 1% relative to the total oil-phase monomer mass, was added to the emulsion. Radical polymerization was conducted at 70 °C and 200 rpm under gentle stirring for 4 h. After completion of the reaction, the product was centrifuged at 8000 rpm for 15 min to collect the microcapsules, followed by vacuum drying at 35 °C for 24 h to obtain the final product, 16-MMWS-K.

#### 2.2.2. Preparation of 16-MMWS-K Microcapsule-Modified Cement Composite Materials

The preparation procedure for the cement composite materials was as follows: At room temperature (~25 °C), Lushan cement, 16-MMWS-K microcapsules, and deionized water were mixed according to the proportions listed in Table 1. Based on the cement mass, the dosages of 16-MMWS-K microcapsule were 0%, 10%, 20%, and 30%. The corresponding composites were designated as 16-MMWS-K-0, 16-MMWS-K-10, 16-MMWS-K-20, and 16-MMWS-K-30. Please note that experiments at a 40% dosage could not be conducted because specimens at this loading failed to maintain structural integrity during demolding and were highly prone to breakage.

The preparation flowchart is shown in Figure S1, and the detailed steps are as follows. First, the 16-MMWS-K microcapsules were soaked in deionized water for 24 h to ensure complete wetting. Subsequently, 500 g of cement was mixed with 1250 g of deionized water to prepare the cement paste [43]. The pre-wetted microcapsules were then weighed according to a predetermined cement mass ratio and added to the cement paste. After thorough mixing, the mortar was cast in layers into a 40 × 40 × 160 mm^3^ mold. Each layer was compacted on a vibrating table (frequency: 2800–3000 cycles/min; amplitude: 0.75 ± 0.02 mm) for 60 ± 5 s, followed by surface finishing. The molded specimens were cured for 24 h in a standard curing chamber at 20 ± 1 °C and relative humidity ≥ 95%. After demolding, specimens were immediately transferred to a constant temperature and humidity curing chamber at 20 ± 1 °C and relative humidity ≥ 95% and cured until the specified age.

### 2.3. Characterization and Testing

Detailed information on the characterization methods and performance measurements is provided in the Supplementary Materials.

## 3. Results and Discussion

### 3.1. Microstructure of 16-MMWS-K Microcapsules

**Figure 2:**
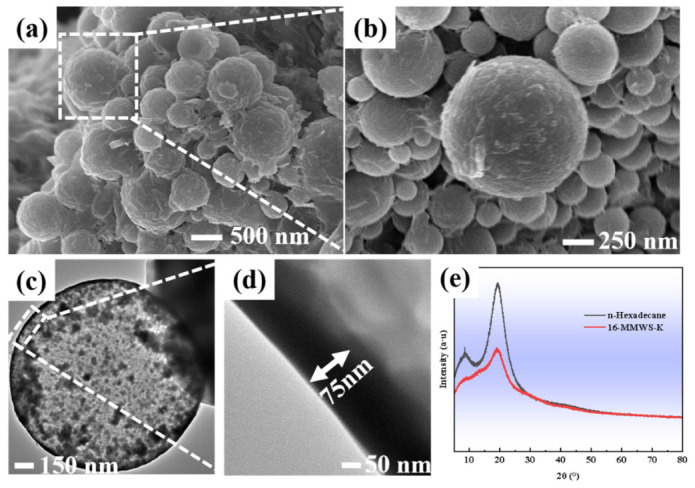
(**a**) Scanning electron microscope image of 16-MMWS-K microcapsule. (**b**–**d**) Transmission electron microscope images of 16-MMWS-K microcapsules recorded at different magnifications. (**e**) XRD patterns of n-n-hexadecane and 16-MMWS-K microcapsules.

Figure 2a shows the morphology and microstructure of the 16-MMWS-K microcapsules. The microcapsules exhibit a regular spherical morphology with excellent dispersion and no noticeable agglomeration, indicating that the SDS emulsification system used in the emulsion polymerization process effectively reduced the oil–water interfacial tension, enabling uniform encapsulation of n-hexadecane [43,44]. The microcapsules are spherical with an average diameter of 250–500 nm. Compared with bare n-hexadecane, which lacks a defined morphology, the microcapsules demonstrate significantly improved size uniformity. Minor wrinkling can be observed on the microcapsule surface, resulting from shrinkage of the PMMA–MBA copolymer shell during polymerization. Following radical polymerization at 70 °C, cooling-induced internal stresses between the polymer shell and n-hexadecane core develop, owing to differences in thermal expansion coefficients. The relatively rigid PMMA segments fail to fully relax these stresses through deformation, ultimately forming surface wrinkles. Transmission electron microscopy (TEM) images of the 16-MMWS-K microcapsules are shown in Figure 2b–d. The TEM images clearly reveal a typical core–shell bilayer structure, where the dark outer layer represents the PMMA-MBA copolymer shell and the lighter inner layer represents the n-hexadecane core. The shell exhibits a well-defined outline with uniform thickness, measured to be approximately 75 nm on average.

Comparison of the X-ray diffraction (XRD) patterns of n-hexadecane and 16-MMWS-K microcapsules (Figure 2e) shows that the microcapsules retained the fundamental crystalline characteristics of n-hexadecane after encapsulation. Pure n-hexadecane exhibits sharp diffraction peaks at 2θ = 19.23°, corresponding to its ordered alkane crystal structure [45,46]. In the XRD pattern of the 16-MMWS-K microcapsules, these characteristic peaks are visible but with significantly reduced intensity and broadened peak shapes, indicating a decrease in crystallinity of the n-hexadecane core owing to spatial confinement by the shell. No new diffraction peaks are observed, confirming the amorphous structure of the PMMA-based shell and showing that the crystalline structure of n-hexadecane is preserved. These results demonstrate that the core material retained its crystalline structures within the microcapsule, indicating excellent structural compatibility between the core and shell materials.

### 3.2. Microstructure of 16-MMWS-K Microcapsule-Incorporated Cement Composites

**Figure 3:**
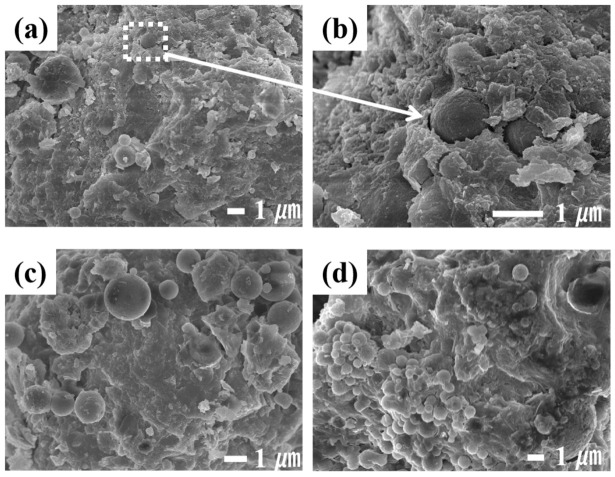
Scanning electron microscope images of three cement composite materials containing (**a**) 10%, (**c**) 20%, and (**d**) 30% 16-MMWS-K microcapsules. (**b**) Enlarged image showing 16-MMWS-K microcapsules embedded in cement matrix.

Figure 3 shows scanning electron microscope images of the 16-MMWS-K-10, 16-MMWS-K-20, and 16-MMWS-K-30 cement composites. As shown in Figure 3a–d, the 16-MMWS-K microcapsules maintain their spherical structure and are uniformly distributed within the cement hydration products. The SEM image in Figure 3b shows that most microcapsules are embedded in the cement matrix with intimate contact, indicating effective mechanical interlocking and some degree of chemical affinity between the PMMA-MBA shell and the C-S-H phase. However, occasional interfacial gaps are also observed, which may arise from shrinkage of the polymer shell during curing and contribute to the increased porosity of the composites. The surface properties and chemical composition of the PMMA-based shell facilitate cement particle adhesion, resulting in the formation of a stable bonding structure. As the microcapsule content increases from 10% to 30%, the number of spherical microcapsules within the matrix increases correspondingly, with a significant improvement in their distribution density. This visually represents the loading and distribution state of microcapsules within the composite material, providing a robust basis for further investigation of the structure–property relationships in microcapsule–cement composites. The observed interfacial bonding is attributed to hydrogen bonding and van der Waals interactions between the ester groups of the PMMA-MBA shell and the Ca^2+^/silanol groups on the surface of C-S-H hydration products, as previously reported for polymer-modified cementitious systems [47,48,49].

### 3.3. Thermal Stability of 16-MMWS-K Microcapsules and Cement Composites

**Figure 4:**
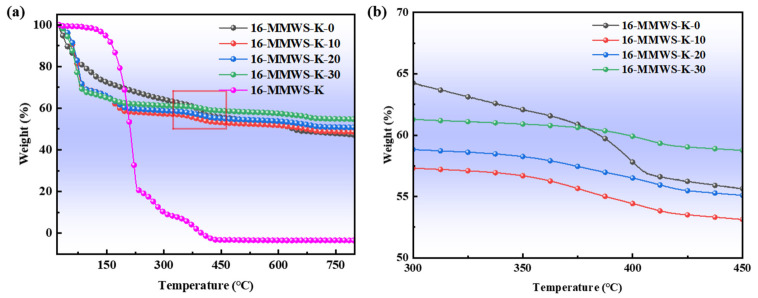
Results of thermogravimetric analysis of 16-MMWS-K cement composites with varying 16-MMWS-K contents in the temperature ranges of (**a**) 100–800 °C and (**b**) 300–450 °C.

Figure 4a,b present the thermal degradation curves of the 16-MMWS-K microcapsules and cement composites containing varying amounts of 16-MMWS-K. At 100 °C, all composites incorporating 16-MMWS-K microcapsules exhibit minor mass loss, likely due to the evaporation of free water during material curing [50]. Within the 100–300 °C temperature range, further mass reduction occurs due to cleavage of C–C bonds in the poly(methyl methacrylate-N,N-methylenebisacrylamide) backbone of the microcapsule shell, producing volatile products, such as carbon dioxide and water. Notably, in the temperature range of 375–425 °C, pure cement specimens experience significant weight loss due to the decomposition of the hydration product Ca(OH)_2_ and the release of various forms of bound water. Conversely, the cement composites incorporating microcapsules exhibit markedly reduced weight loss during this temperature range [51]. This improvement can be attributed to the dense microcapsule shell, which increases the tortuosity of the diffusion pathways for small molecules, such as water vapor released during the decomposition of hydration products, thereby delaying their escape. Additionally, the presence of microcapsules reduces the proportion of thermally decomposable hydration products per unit mass of the composite, producing a diluting effect. Furthermore, the phase transition of n-hexadecane in the microcapsule core absorbs heat, whereas the thermal decomposition of the shell polymer consumes substantial heat, collectively reducing the local heating rate and temperature within the cement matrix. These combined effects enhance the overall thermal stability of the hydration products. Consequently, the composite material exhibits a relatively moderate weight-loss behavior within the temperature range of 375–425 °C.Notably, the weight loss at high temperatures (e.g., >400 °C) should not be interpreted as melting of the microcapsules. The PCM core melts at ~15 °C, while both the core and the PMMA-MBA shell decompose completely below 500 °C, leaving only inorganic residues at 750 °C.

### 3.4. Phase Transition Characteristics of 16-MMWS-K and Cement Composite Materials

**Figure 5:**
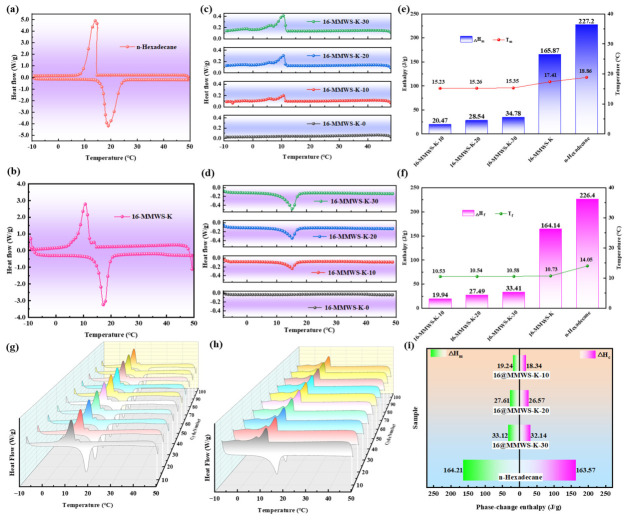
DSC curves of (**a**) n-hexadecane, (**b**) 16-MMWS-K microcapsules, and (**c**,**d**) cement composites with varying 16-MMWS-K contents. (**e**) Melting and (**f**) crystallization temperatures, along with corresponding enthalpies of 16-MMWS-K microcapsules, n-hexadecane, and cement composites with different 16-MMWS-K contents. (**g**,**h**) DSC cycles of 16-MMWS-K microcapsules and 16-MMWS-K-30. (**i**) Enthalpy after cycling.

The 16-MMWS-K microcapsules were incorporated into cement to prepare phase-change energy storage cement composites, which were then subjected to differential scanning calorimetry (DSC) testing. As shown in Figure 5g–i, Figures S2 and S3, melting temperatures of the cement composites range from 15.2 to 15.4 °C. The melting temperatures for composites with 10%, 20%, and 30% incorporation are determined as 15.23 °C, 15.26 °C, and 15.35 °C, respectively. The observed melting temperature range closely matches the indoor thermal comfort range of buildings, making it suited for compliance with building energy regulations. The crystallization temperatures of the composites are concentrated in the range of 10.5–10.6 °C, with corresponding values of 10.53 °C, 10.54 °C, and 10.58 °C for the samples containing 10%, 20%, and 30% microcapsules, respectively. The melting enthalpy and crystallization enthalpy of pure n-hexadecane are 227.20 J/g and 226.40 J/g, respectively, whereas the corresponding values for the 16-MMWS-K microcapsules are 165.87 J/g and 164.14 J/g, respectively.

The encapsulation efficiency was calculated using the following equation [52]:

(1) $$E=\frac{\Delta H_{m,16-\mathrm{MMWS}-K}+\Delta H_{f,16-\mathrm{MMWS}-K}}{\Delta H_{m,n-\mathrm{Hexadecane}}+\Delta H_{f,n-\mathrm{Hexadecane}}}\times 100\%$$

Based on this calculation, the encapsulation efficiency is determined to be 72.75%. As the dosage of 16-MMWS-K microcapsules increases, the phase-change latent heat of the cement composites exhibit a monotonic increasing trend. For the 16-MMWS-K-30 sample with 30% microcapsules, the melting enthalpy (ΔH_m_) and crystallization enthalpy (ΔH_f_) reach 34.78 J/g and 33.41 J/g, respectively. As shown in Figure 5g–i, Figures S2 and S3, the enthalpy after cycling has not significantly decreased compared to before cycling. These results demonstrate that the 16-MMWS-K microcapsules effectively enhances the thermal energy storage capacity of the cement matrix, improving the thermal inertia of building envelopes and their potential for regulating indoor thermal environments.

### 3.5. Thermal Conductivity and Thermal Imaging Analysis of Cement Composites

**Figure 6:**
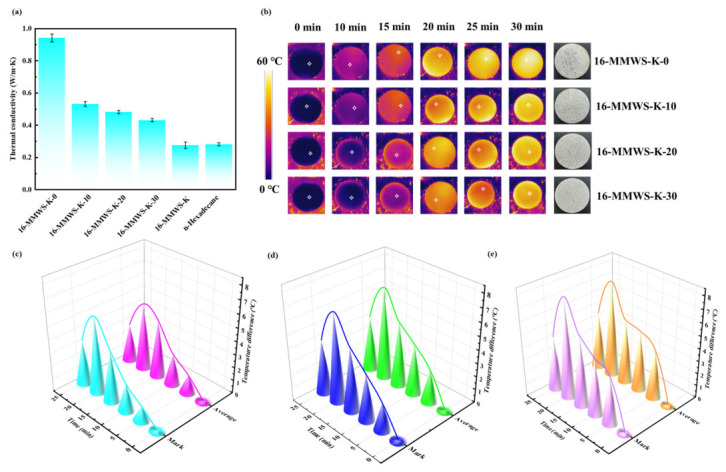
(**a**) Thermal conductivity of 16-MMWS-K microcapsules, n-hexadecane, and cement-based composites with varying 16-MMWS-K contents, along with relative reduction compared with that of 16-MMWS-K-0. (**b**) Infrared thermal imaging maps of cement composites with varying 16-MMWS-K contents; (**c**–**e**) Infrared thermal imaging annotations and average temperature difference distribution profiles of 16-MMWS-K-10, 16-MMWS-K-20, and 16-MMWS-K-30 compared with that of 16-MMWS-K-0 at different time points. Each peak represents the spatially averaged temperature at a specific time interval.

The 16-MMWS-K microcapsules were incorporated into cement to prepare phase-change energy-storage cement composites, which were subsequently subjected to thermal conductivity testing. The results are presented in Table S2 and Figure 6a. As the dosage of 16-MMWS-K microcapsules increases, thermal conductivity of the cement composites decreases significantly. The reference group without microcapsule incorporation (16-MMWS-K-0) exhibits a thermal conductivity of 0.9175 W/(m·K). The 16-MMWS-K-10 composite with 10% microcapsules exhibits a thermal conductivity of 0.5147 W/(m·K), which is 43.90% lower compared with that of the reference group. The 16-MMWS-K-20 composite with 20% microcapsules exhibits a thermal conductivity of 0.4712 W/(m·K), achieving a 48.64% relative reduction. The 16-MMWS-K-30 composite with 30% microcapsules exhibits a thermal conductivity of 0.4218 W/(m·K), achieving a 54.03% relative reduction. Pure 16-MMWS-K microcapsules and n-hexadecane exhibit characteristically low thermal conductivities of 0.2832 and 0.2814 W/(m·K), respectively. As the microcapsule content increases from 0% to 30%, thermal conductivity of the composite material systematically decreases from 0.9175 to 0.4218 W/(m·K). At 30% microcapsule content, the thermal conductivity is 54.03% lower than that of the reference group. The low thermal conductivities exhibited by the pure 16-MMWS-K microcapsules and n-hexadecane indicate that the microcapsules function as an effective thermal suppression phase [53]. These results demonstrate that incorporating 16-MMWS-K microcapsules significantly reduces the thermal conductivity of cement composites, thereby enhancing thermal insulation performance and improving the energy storage efficiency of phase change materials for thermal management of buildings.

Thermal differential analysis based on infrared thermography (Figure 6b–e) indicates that incorporating 16-MMWS-K microcapsules effectively delays the temperature rise of the cement matrix, with the thermal regulation efficacy positively correlating with microcapsule dosage. During the initial heating phase (0–5 min), the central temperature difference between the 30% microcapsule-incorporated specimen and the reference specimen reaches 3.8 °C, which is significantly higher than those of the 10% and 20% specimens, indicating more pronounced thermal insulation effects at higher dosages. During the phase transition stage (10–20 min), the temperature difference of the 30% dosage sample plateaus at 10 min, reflecting extensive heat absorption by the n-hexadecane core material, which effectively suppresses the temperature rise. By 20 min, the core temperature difference further increases to 7.9 °C, highlighting optimal thermal buffering capacity [54]. During the initial cooling phase (25 min), the 30% dosage sample maintains the highest temperature difference, indicating that the latent heat release effectively slows the temperature decline. These results demonstrate that 16-MMWS-K microcapsules significantly enhance the thermal inertia of cementitious materials through phase-change behavior, with higher dosage systems exhibiting superior temperature regulation performance throughout the thermal cycle.

### 3.6. Hydrophilic and Hydrophobic Testing of Cement Composites

**Figure 7:**
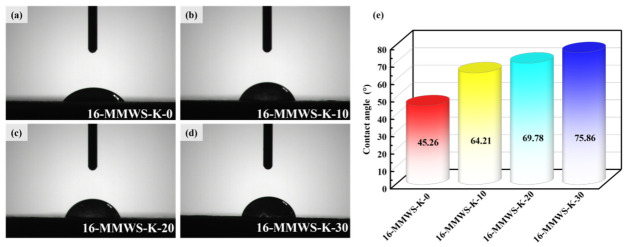
(**a**–**d**) Contact angle images and (**e**) contact angles of cement composites with varying 16-MMWS-K contents.

To systematically characterize the regulation of surface hydrophilic/hydrophobic properties of cement composites using 16-MMWS-K microcapsules at different mass fractions, water contact angle tests were conducted on specimens with varying microcapsule contents. The results presented in Table S3 and Figure 7a–e show a monotonic increase in contact angle with increasing microcapsule content. The unmodified reference sample (16-MMWS-K-0) exhibits a water contact angle of only 45.26°. Owing to the abundance of hydrophilic hydroxyl groups (-OH) on the cement matrix surface, 16-MMWS-K-0 exhibits strong molecular polarity and high water adsorption capacity, demonstrating a typical strong hydrophilicity. Incorporation of 10% microcapsules increases the contact angle of 64.21°, representing a 41.87% increase compared with that of the reference sample. This increase in water contact angle arises from the hydrophobic nature of the PMMA shells of the microcapsules. The PMMA shells form an initial continuous coating layer on the cement matrix surface, effectively shielding the hydrophilic hydroxyl groups and reducing their exposure, thereby inducing a phase transformation in surface wettability. With further increases in microcapsule content, the contact angle continues to increase but at a slower rate: at 20% incorporation, the contact angle reaches 69.78%, and at 30% incorporation, 75.86%, corresponding to a total relative increase of 67.61% compared with that of the reference sample, achieving the optimal hydrophobic state. This trend occurs because the fundamental construction of the hydrophobic layer—“from nothing to something”—is essentially completed at the 10% loading stage, and subsequent increases reinforce and optimize the PMMA coverage. With a stable microcapsule dispersion, the probability of water molecules contacting the cement hydroxyl groups gradually decreases, causing the percentage increase in contact angle to decrease from approximately 41.87% to 8%.

These quantitative results provide direct experimental evidence that surface-wetting properties of cementitious materials can be tailored to meet requirements, such as moisture resistance and waterproofing in construction applications. The observed changes in contact angle changes further confirm the feasibility and effectiveness of regulating the hydrophilicity and hydrophobicity of cementitious materials using hydrophobic polymer-coated microcapsules.

### 3.7. Thermal Energy Storage in Buildings

**Figure 8:**
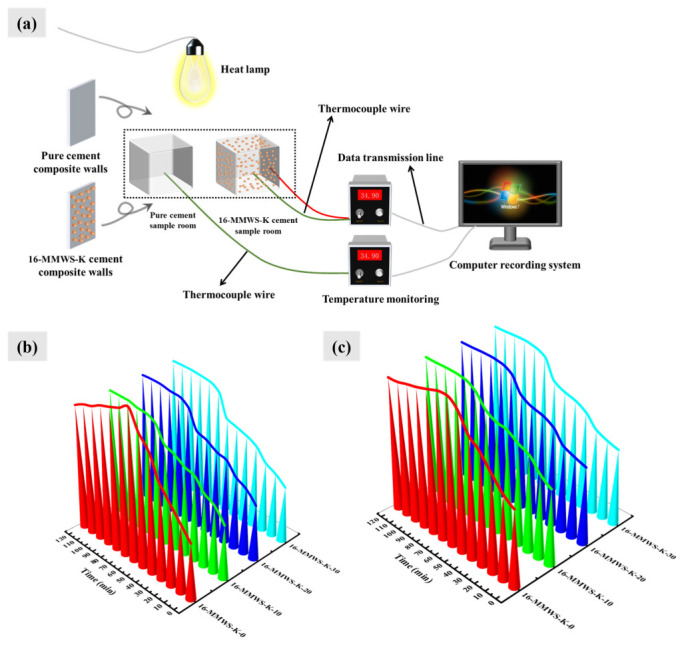
(**a**) Schematic of apparatus used to evaluate thermal energy storage capacity of 16-MMWS-K microcapsule-reinforced cement composites. Comparison of thermal regulation performance between pure cement and 16-MMWS-K cement composite materials in a simulated building: (**b**) wall temperature changes and (**c**) central temperature changes with time.

To evaluate the thermal energy storage performance of the 16-MMWS-K microcapsule cement composite, it was installed within the wall structure of a custom model room, as shown in Figure 8a. The test cycle comprised a 60-min heating phase, followed by a 60-min natural cooling phase. The temperature changes at the room center and wall surface were recorded every 10 min (Figure 8b,c and Tables S4 and S5). When exposed to heat, the composite walls incorporating 16-MMWS-K microcapsules exhibit a pronounced thermal response lag, significantly outperforming conventional cement walls without microcapsules. During the initial heating phase (0–30 min), the central temperature of the composite wall increases slowly because the n-hexadecane core within the microcapsules has not yet undergone phase transition, and its low thermal conductivity delays heat transfer. As heating continues, n-hexadecane begins to melt and absorb latent heat, effectively suppressing indoor temperature increase [51]. After 60 min of heating, the central temperature of the 16-MMWS-K-30 sample reaches only 25.3 °C, whereas that of the reference group (16-MMWS-K-0) reaches 32.7 °C, resulting in a temperature difference of 7.4 °C. The surface temperature difference is even more pronounced, with the 16-MMWS-K-30 sample at 26.8 °C and the reference group at 38.7 °C, showing a maximum difference of 11.9 °C. During the subsequent 60-min natural cooling period, the microcapsule-reinforced wall releases stored latent heat through n-hexadecane crystallization, effectively slowing indoor temperature decline. At the end of cooling (total duration: 120 min), the central temperature of the 16-MMWS-K-30 sample reaches 31.9 °C, which is 4.3 °C higher than that of the reference group. Note that the temperature of the 16-MMWS-K-30 sample wall (34.4 °C) also exceeds that of the reference group. These results demonstrate that 16-MMWS-K microcapsules effectively absorb and store heat during heating, delaying the temperature, and gradually releasing heat during cooling, slowing temperature drop and significantly stabilizing indoor temperature fluctuations. With a suitable phase transition temperature and excellent thermal stability, the 16-MMWS-K microcapsule cement composite shows great potential for enhancing the thermal inertia of building envelopes and optimizing indoor thermal environments. This contributes to reducing heating and cooling energy consumption in buildings while improving energy utilization efficiency.

### 3.8. Mechanical Properties of Cement Composite Materials

**Figure 9:**
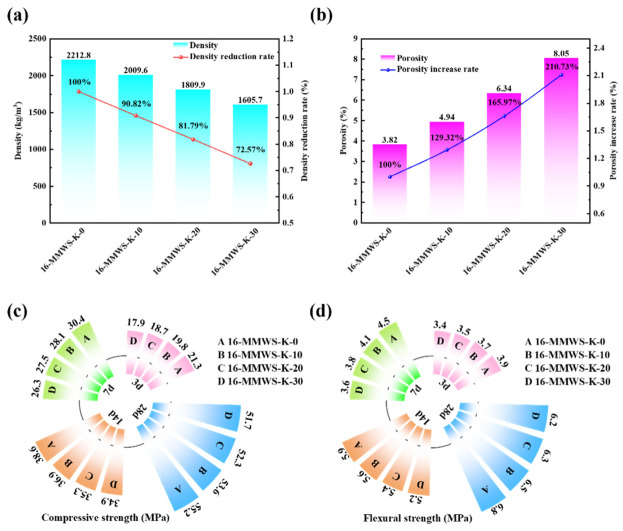
(**a**) Density and (**b**) porosity of cement composites with varying 16-MMWS-K microcapsule contents. (**c**) Compressive strength and (**d**) flexural strength of cement composites with varying 16-MMWS-K contents over different curing ages.

As shown in Table S5 and Figure 9a,b, the dosage of 16-MMWS-K microcapsules significantly affect the density and porosity of the cement composite material. As the microcapsule content increases from 0% to 30%, density of the composite decreases from 2212.8 kg/m^3^ to 1605.7 kg/m^3^, corresponding to a reduction of 27.43%. Meanwhile, the porosity increases from 3.82% to 8.05%, corresponding to a substantial increase of 110.73%. The decrease in density can be attributed to the inherently low density of the 16-MMWS-K microcapsules. The significant increase in porosity can be attributed to the expansion of the interfacial transition zone volume after microcapsule incorporation.

The effects of microcapsule dosage on mechanical properties of the cement composites are presented in Tables S5–S7, with variation patterns illustrated in Figure 9c,d. At all curing ages (3, 7, 14, and 28 days), both compressive and flexural strengths of the composite materials gradually decrease with increasing microcapsule content. This phenomenon stems primarily from two factors [55,56]. First, the inherently low mechanical strength of PMMA-based microcapsules creates stress concentration points under compressive and flexural loads, weakening the overall load-bearing capacity of the composite. Second, the addition of microcapsules significantly increases porosity (Table S5), further reducing mechanical performance [56,57]. The composites containing 10%, 20%, and 30% microcapsules exhibit 28-day compressive strengths of 53.6 MPa, 52.3 MPa, and 51.7 MPa, respectively, corresponding to decreases of 2.9%, 5.3%, and 6.3%, respectively. Similarly, the composites containing 10%, 20%, and 30% microcapsules exhibit 28-day flexural strengths of 6.8 MPa to 6.5 MPa, 6.3 MPa, and 6.2 MPa, respectively, corresponding to decreases of 4.4%, 7.4%, and 8.8%, respectively. Despite the reductions in mechanical strength, the composite material with the highest microcapsule content (30%) still exhibit a 28-day compressive strength of 51.7 MPa and a flexural strength of 6.2 MPa, both exceeding the minimum requirements for conventional building materials and meeting practical engineering demands. The inverse correlation between porosity and mechanical strength is evident from Table S5: as microcapsule content increases from 0% to 30%, porosity rises from 3.82% to 8.05%, accompanied by a 6.3% reduction in compressive strength and an 8.8% reduction in flexural strength at 28 days, indicating that pore structure deterioration is the primary cause of mechanical degradation.

To further enhance the mechanical performance of cement composites while preserving their energy storage capacity, future research may focus on incorporating ultrafine mineral admixtures, such as silica fume and nano-calcium carbonate. These additives can refine the interfacial microstructure between 16-MMWS-K microcapsules and the cement matrix, strengthening interfacial bonding and achieving synergistic improvements in both thermal and mechanical properties [58,59,60,61].

## 4. Conclusions

In this study, 16-MMWS-K microcapsules with a crosslinked structure were successfully synthesized via emulsion polymerization, and their potential applications as phase-change energy-storage functional components in cement composites were systematically investigated. The results indicate that the synthesized 16-MMWS-K microcapsules exhibit a well-defined spherical core–shell structure, excellent thermal stability, and suitable phase-change temperatures (melting temperature of ~15.3 °C and crystallization temperature of ~10.6 °C). With a coating efficiency of 72.75%, leakage of the PCM core was effectively suppressed while preserving the crystallization behavior of the core material.

After incorporation of the 16-MMWS-K microcapsules into the cement matrix, the thermal properties of the composite material were significantly enhanced. With increasing microcapsule content, the latent heat storage capacity of the composite markedly improved, with the melting enthalpy reaching 34.78 J/g oat a microcapsule dosage of 30%. Meanwhile, the thermal conductivity decreased by 54.03%, indicating excellent heat storage and thermal insulation performance. Infrared thermal imaging and building simulation experiments further confirmed that microcapsule-reinforced walls exhibited a pronounced temperature-lag effect during thermal cycling, effectively delaying indoor temperature fluctuations and enhancing the thermal inertia of the building. Additionally, the incorporation of microcapsules significantly enhanced the hydrophobicity of the cement matrix. At a 30% dosage, the contact angle increased to 75.86°, indicating improved moisture resistance and material durability. Although microcapsule addition increased the porosity of the cement composites and caused some reduction in compressive and flexural strength, the 28-day compressive strength at a 30% dosage still reached 51.7 MPa, meeting the basic mechanical requirements for structural building materials. Although the literature has confirmed the chemical stability of PMMA-based microcapsules in alkaline cement environments [62,63,64,65,66], and the SEM, TGA, and DSC cycling results from this study indirectly support their structural integrity, quantitative long-term durability assessments—including prolonged alkali erosion and long-term thermal cycling—remain an important focus of our future research to further validate their potential for engineering applications. While the thermal performance evaluation is based on a laboratory-scale model, the results demonstrate the temperature-regulating capability of the composites. Real-world applications will require systematic evaluation of wall design, climate conditions, and building integration in future studies. Preliminary economic analysis indicates that the additional material cost can be partially offset by reduced operational energy consumption, with further improvements expected from scale-up production.

## Figures and Tables

**Table 1:** Composition of 16-MMWS-K microcapsule-reinforced cement composites.

Sample	Water (g)	Cement (g)	16-MMWS-K Microcapsule (g)
16-MMWS-K-0	500	1250	0
16-MMWS-K-10	500	1250	125
16-MMWS-K-20	500	1250	250
16-MMWS-K-30	500	1250	375

## Data Availability

The original contributions presented in this study are included in the article and supplementary material. Further inquiries can be directed to the corresponding authors.

## Supplementary Materials

The following supporting information can be downloaded at: https://www.mdpi.com/article/10.3390/polym18131609/s1, Figure S1: Preparation of 16-MMWS-K icrocapsule-reinforced cement composite materials; Figure S2: DSC diagram after 16-MMWS-K-20 cycle; Figure S3: DSC diagram after 16-MMWS-K-10 cycle; Table S1: Melting and crystallization temperatures and enthalpy values of 16-MMWS-K, n-N-Hexadecane, and cement composites with varying 16-MMWS-K contents; Table S2: Thermal Conductivity of 16-MMWS-K, n-N-Hexadecane, and Cement Composites with Different 16-MMWS-K Content and Relative Reduction Compared to 16-MMWS-K-0; Table S3: Contact angle values and derived quantitative metrics corresponding to different 16-MMWS-K microcapsule content levels; Table S4: Thermal imaging annotations and average temperature differences for 16-MMWS-K-10, 16-MMWS-K-20, 16-MMWS-K-30, and 16-MMWS-K-0 at different time points; Table S5: Specific Temperature Values at Wall Surface and Room Center for Pure Cement and 16-MMWS-K Composite Wall Panels; Table S6:Density and porosity of cement composites with different 16-MMWS-K Microcapsule Content; Table S7: Comparison of compressive strength of cement composites with different 16-MMWS-K contents; Table S8:Comparison of flexural strength in cement composites with different 16-MMWS-K content.
