# Supplementary material for: Preparation, Thermal Regulation, and Energy Storage Properties of n-hexadecane@polymethyl Methacrylate Microcapsule–Cement Composite Phase Change Materials
Source: Polymers (Basel). 2026 Jun 28;18(13):1609. doi: 10.3390/polym18131609 (PMC13363734; doi:10.3390/polym18131609)
Supplement: Supplementary file 1 [file polymers-18-01609-s001.zip › polymers-4381452-supplementary.pdf]

Supporting information

Preparation, thermal regulation, and energy storage properties of n-hexadecane@polymethyl methacrylate microcapsule–cement composite phase change materials

Houqi Zhu[a], Jianmin Ma[a], Xiaoxiao Xing[a], Yuting He[a], Heng Wang[b], Lixian Sun[a] , Cuili Xiang[a,]*and Yongjin Zou[a,]*

[a]*College of Materials Science and Engineering, Guilin University of Electronic Technology, Guilin 541004, China*

[b]*Guilin Huayue Environmental Protection Technology Co., Ltd., Guilin 541805, China*

*Corresponding authors:

Cuili Xiang

College of Materials Science and Engineering, Guilin University of Electronic Technology, Guilin 541004, China

E-mail: xiangcuili@guet.edu.cn

Yongjin Zou

College of Materials Science and Engineering, Guilin University of Electronic Technology, Guilin 541004, China

E-mail: zouy@guet.edu.cn

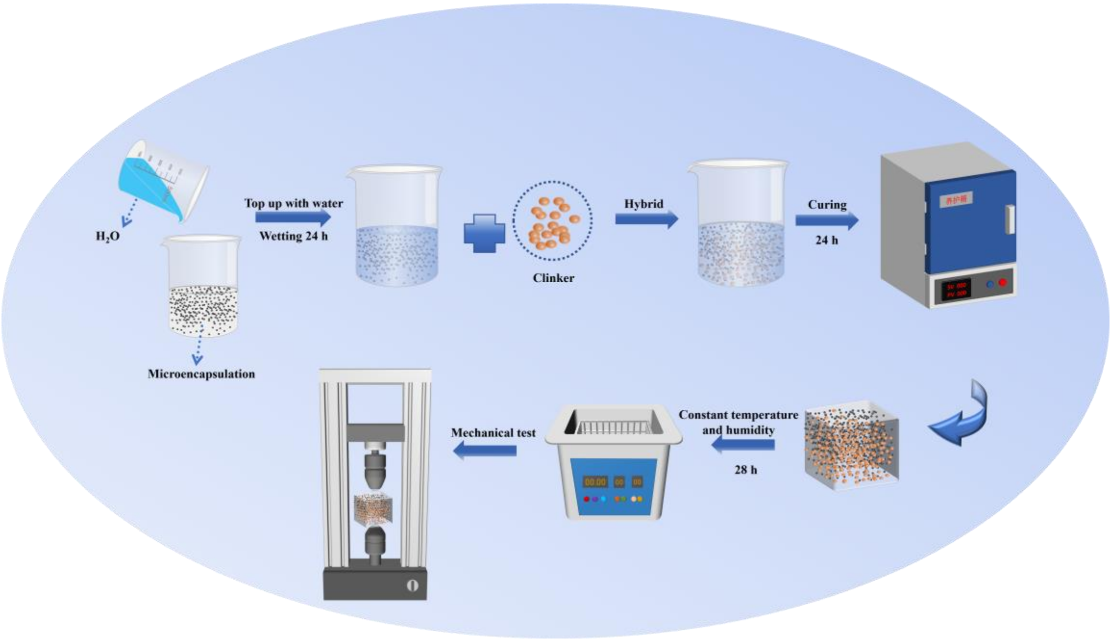

Fig. S1. Preparation of 16-MMWS-K microcapsule-reinforced cement composite materials.

Characterizations and measurements

The morphology, size, and microstructure of the 16-MMWS-K microcapsule/cement composite were characterized using a scanning electron microscope (SEM, SU8020 model) at an acceleration voltage of 1.0 kV. All samples underwent gold sputtering treatment prior to testing. The shell wall thickness of the microcapsules was further determined using a transmission electron microscope (TEM, FEI Tecnai G2 F20 model). Additionally, XRD patterns of 16-MMWS-K microcapsules and n-hexadecane were collected using a SmartLab 9 kW X-ray diffractometer (equipped with a Cu Kα radiation source, operating voltage 40 kV, current 40 mA). The scanning range was 10°–80° (2θ) at a scanning rate of 3°/min.

The enthalpy values and phase transition characteristics of 16-MMWS-K microcapsule-cement composites were investigated using differential scanning calorimetry (DSC; DSC 250). Under a nitrogen flow rate of 285 mL/min, the

measurement temperature was raised from ambient to 50°C at a heating rate of 5°C/min, then lowered from 50°C to -10°C, and finally raised again from -10°C to 50°C. Thermogravimetric analysis (TGA) of the 16-MMWS-K microcapsule-cement composite was performed using a TA Q50 thermogravimetric analyzer under a nitrogen atmosphere. Samples were heated from 30°C to 600°C at a rate of 5°C/min. The thermal conductivity of the 16-MMWS-K microcapsule-cement composite was determined using an HS-DR-5 thermal conductivity analyzer.

To evaluate the thermal control performance of cement-based composites, thermal behavior testing was conducted on specimens using an infrared thermal imaging camera (E09). Cement discs (diameter 25 mm, thickness 3 mm) containing 10%, 20%, and 30% 16-MMWS-K were prepared, with pure cement specimens without microcapsules serving as the control group. All specimens were pre-cooled to 0°C before being placed on a 60°C constant-temperature heating platform. To slow the heating rate, circular polytetrafluoroethylene (PTFE) insulation discs (diameter 25 mm, thickness 5 mm) were positioned between the heating plate and the specimens. During testing, the thermal imaging camera lens maintained a 25-centimeter distance from the specimen's upper surface. Infrared thermal images were captured every 5 minutes within the 0-25 minute range for subsequent analysis.

The energy storage performance of the 16-MMWS-K microcapsule-cement composite material was evaluated using a customized testing system (Fig. S2) [43-44]. This apparatus employed a xenon lamp (CEL-HXF300-T3; Beijing Saileite Technology Co., Ltd.) as the heat source and featured two independent chambers: one for pure cement and another for the 16-MMWS-K microcapsule-cement composite panel. This configuration enabled direct comparison between the control and composite samples under identical conditions. Temperature data were collected via thermocouples

connected to the monitoring system and transmitted via data cables to a computer data logging interface for real-time recording. The test procedure comprised a 60-minute heating phase followed by a 60-minute natural cooling phase, with temperature values recorded every 10 minutes. To establish a baseline, cement boards of identical dimensions were tested under the same conditions as the control group.

This study employed an Auto Pore IV 9605 mercury porosimeter (MIP) to determine the pore structure of cement composites under pressures reaching 33,000 psi.

The mechanical properties of 16-MMWS-K microcapsule-cement composites at different mixing ratios were evaluated through compressive strength tests conducted at 3, 7, 14, and 28 days. The tests employed cubic specimens measuring 40 × 40 × 160 mm^3.

Table S1. Melting and crystallization temperatures and enthalpy values of 16-MMWS-K, n-N-Hexadecane, and cement composites with varying 16-MMWS-K contents.

Name	Melting temperature (°C)	Enthalpy of fusion $\triangle H_m$ (J/g)	Crystallization temperature (°C)	Crystallization enthalpy $\triangle H_f$ (J/g)
16-MMWS-K-0	-	-	-	-
16-MMWS-K-10	15.23	20.47	10.53	19.94
16-MMWS-K-20	15.26	28.54	10.54	27.49
16-MMWS-K-30	15.35	34.78	10.58	33.41
16-MMWS-K	17.41	165.87	10.73	164.14

n-Hexadecane	18.86	227.20	14.05	226.40

Table S2. Thermal Conductivity of 16-MMWS-K, n-N-Hexadecane, and Cement Composites with Different 16-MMWS-K Content and Relative Reduction Compared to 16-MMWS-K-0.

Name	Thermal conductivity	Relative reduction in volume compared to 16-MMWS-K-0 (%)
16-MMWS-K-0	0.9175	-
16-MMWS-K-10	0.5147	43.90
16-MMWS-K-20	0.4712	48.64
16-MMWS-K-30	0.4218	54.03
16-MMWS-K	0.2832	69.13
n-Hexadecane	0.2814	69.33

Table S3. Contact angle values and derived quantitative metrics corresponding to different 16-MMWS-K microcapsule content levels.

Name	Water contact angle (°)	Relative increment compared to 16-MMWS-K-0 (%)	Compared to the previous batch, the month-over-month growth rate (%)
16-MMWS-K-0	45.26	-	-

16-MMWS-K-10	64.21	41.87	41.87
16-MMWS-K-20	69.78	54.18	8.68
16-MMWS-K-30	75.86	67.61	8.71

Table S4. Thermal imaging annotations and average temperature differences for 16-MMWS-K-10, 16-MMWS-K-20, 16-MMWS-K-30, and 16-MMWS-K-0 at different time points.

Temperature difference (°C) / Time (min)	16-MMWS-K-10		16-MMWS-K -20		16-MMWS-K-30	
	Mark	Average	Mark	Average	Mark	Average
0	0	0	0	0	0	0
5	1.1	1.4	2.1	2.5	3.8	3.9
10	2.1	2.4	3.5	3.8	4.5	4.8
15	3.9	4.4	4.8	4.9	5.6	5.1
20	5.8	5.1	6.7	6.9	7.9	7.6
25	3.4	2.9	4.1	4.2	4.3	4.6

Table S5. Specific Temperature Values at Wall Surface and Room Center for Pure Cement and 16-MMWS-K Composite Wall Panels

Temperature (°C) / Time (min)	16-MMWS-K-0		16-MMWS-K -10		16-MMWS-K-20		16-MMWS-K-30	
	Center	Wall	Center	Wall	Center	Wall	Center	Wall
0	17.7	14.8	17.4	14.3	17.8	14.6	17.6	14.8
10	19.5	18.2	19.2	17.9	18.7	17.6	18.4	17.1
20	22.4	21.6	22.1	19.5	19.8	19.2	19.6	18.7
30	25.5	25.1	23.3	22.7	22.9	22.4	22.4	21.9
40	27.6	31.4	24.9	23.6	24.3	23.2	23.9	23.4
50	31.8	34.7	25.4	25.3	25	24.9	24.7	24.4
60	33.6	39.6	26.8	26.4	26.4	25.9	26	25.3
70	33.4	38.1	29.8	30.8	30.2	31.2	30.6	31.6
80	32.6	37.2	31.2	32.1	31.4	32.8	31.8	33.2
90	31.9	36.4	31.6	32.4	31.8	33.1	32.3	33.7
100	31.4	35.1	31.7	33.1	31.9	33.7	32.4	34.1
110	30.8	34.4	31.7	33.2	31.9	33.9	32.4	34.3

120	30.7	33.1	31.8	33.3	32	33.9	32.5	34.4

Table S6. Density and porosity of cement composites with different 16-MMWS-K Microcapsule Content.

Name	Density (kg/m^3)	Density reduction rate (%)	Porosity (%)	Porosity increase rate (%)
16-MMWS-K-0	2212.8	-	3.82	-
16-MMWS-K-10	2009.6	9.18	4.94	29.32
16-MMWS-K-20	1809.9	18.21	6.34	65.97
16-MMWS-K-30	1605.7	27.43	8.05	110.73

Table S7. Comparison of compressive strength of cement composites with different 16-MMWS-K contents.

Name	3d (MPa)	7d (MPa)	14d (MPa)	28d (MPa)
16-MMWS-K-0	21.3	30.4	38.6	55.2
16-MMWS-K-10	19.8	28.1	36.9	53.6
16-MMWS-K-20	18.7	27.5	35.3	52.3
16-MMWS-K-30	17.9	26.3	34.9	51.7

Table S8. Comparison of flexural strength in cement composites with different

16-MMWS-K content.

Name	3d (MPa)	7d (MPa)	14d (MPa)	28d (MPa)
16-MMWS-K-0	3.9	4.5	5.9	6.8
16-MMWS-K-10	3.7	4.1	5.6	6.5
16-MMWS-K-20	3.5	3.8	5.4	6.3
16-MMWS-K-30	3.4	3.6	5.2	6.2

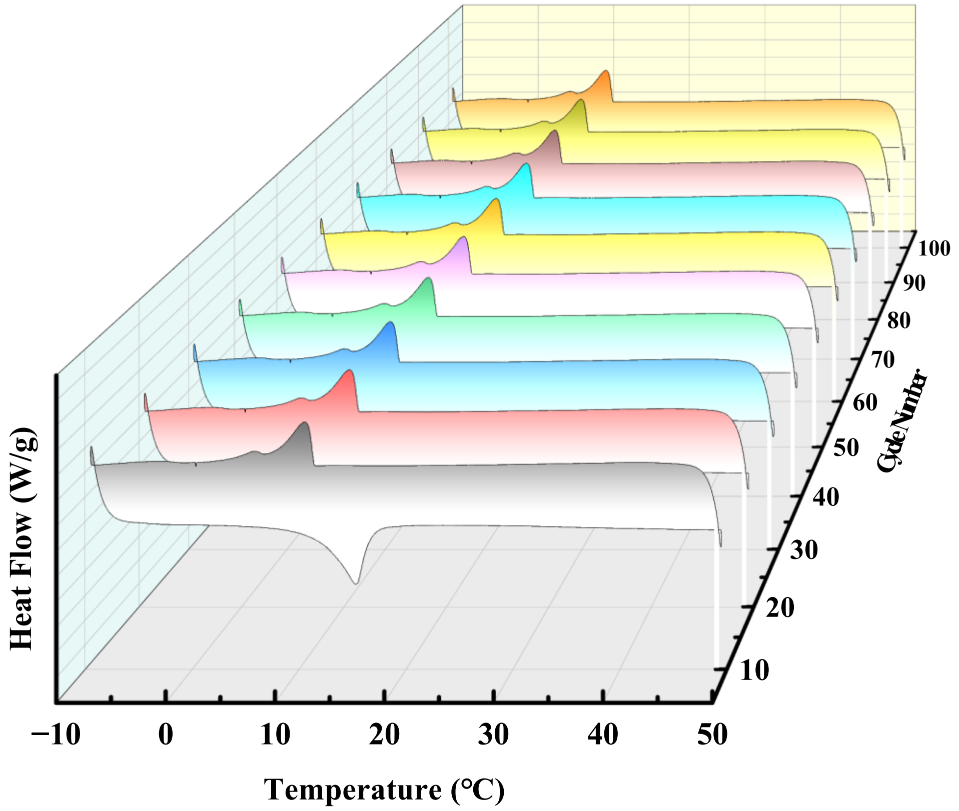

Fig. S2. DSC diagram after 16-MMWS-K-20 cycle

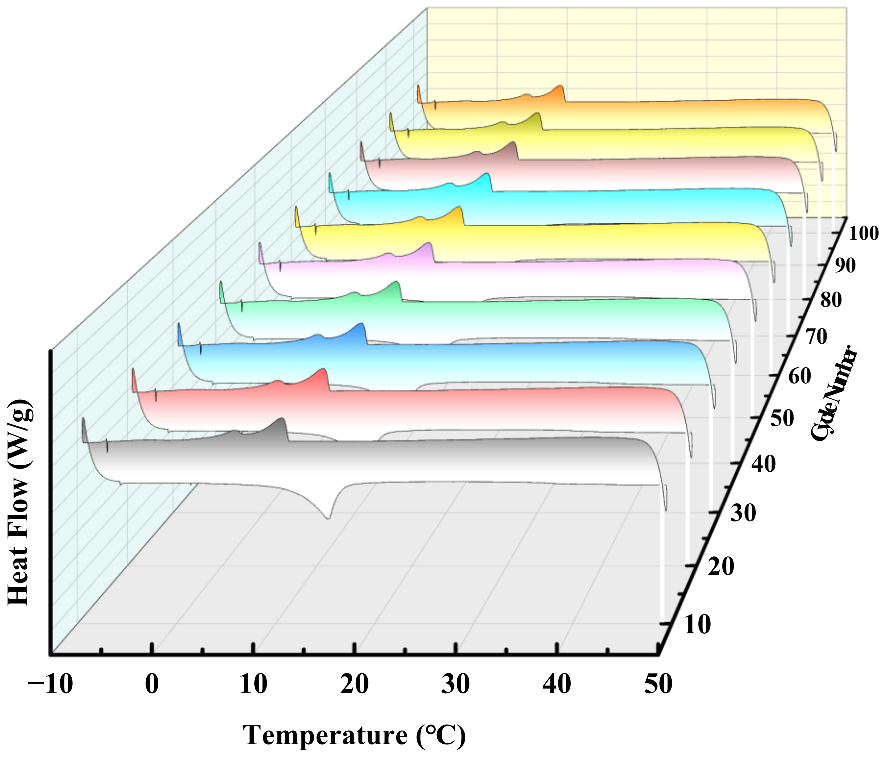

Fig. S3. DSC diagram after 16-MMWS-K-10 cycle
